# Molecular characterization of Fe-acquisition genes causing decreased Fe uptake and photosynthetic inefficiency in Fe-deficient sunflower

**DOI:** 10.1038/s41598-021-85147-z

**Published:** 2021-03-10

**Authors:** Ahmad Humayan Kabir, Sharaban Tahura, Mona M. Elseehy, Ahmed M. El-Shehawi

**Affiliations:** 1 Department of Botany, University of Rajshahi, Rajshahi, 6205 Bangladesh; 2 Department of Genetics, Faculty of Agriculture, Alexandria University Alexandria, Alexandria, Egypt; 3 Department of Biotechnology, College of Science, Taif University, P.O. Box 11099, Taif, 21944 Saudi Arabia

**Keywords:** Plant physiology, Plant stress responses

## Abstract

Iron (Fe) deficiency in plants hinders growth and yield. Thus, this study aims to elucidate the responses and molecular characterization of genes in Fe-deficient sunflower. The study was conducted on 14 days-old sunflower plants cultivated in hydroponic culture under Fe-sufficient and Fe-deficient conditions. The Fe-starved sunflower showed substantial decrease in plant biomass, SPAD score, quantum yield efficiency of PSII (Fv/Fm), photosynthetic performance index (Pi_ABS). Further, Fe shortage reduced Fe and Zn concentrations in roots and shoots, accompanied by a marked decrease of *HaNramp1* and *HaZIP1* expression in roots, suggesting the association of Zn status contributing to photosynthetic inefficiency in sunflower. The ferric chelate reductase (FCR) activity, along with *HaFRO2* and *HaIRT1* transcripts, were constitutively expressed, suggesting that sunflower plants can regulate FCR activity, although the lack of bioavailable Fe in the rhizosphere strongly corresponds to the limited Fe uptake in sunflower. The substantial increase of proton extrusion in roots and the localization of Fe-related genes in the plasma membrane are also evident in sunflower as common responses to Fe-deficiency by this Strategy I plant species. Analysis showed that three motifs of Fe-related proteins were linked to the *ZIP* zinc transporter. The interactome map revealed the close partnership of these Fe-related genes in addition to *FRU* gene encoding putative transcription factor linked to Fe uptake response. The *cis*-regulatory analysis of promoter suggested the involvement of auxin, salicylic acid, and methyl jasmonate-responsive elements in the regulatory process in response to Fe deficiency. These findings may be beneficial to develop Fe-efficient sunflower plants through breeding or genome editing approaches.

## Introduction

Iron (Fe) deficiency negatively affects the growth and yield of plants. Fe-deficiency causes a stunted root growth and poor maturation of fruits^1,2^. In alkaline soil, the problem most commonly occurs due to low Fe solubility at high pH^1,3^. In plants, photosynthesis, respiration, and protein formation are closely related to the Fe status^4,5^. In the photosynthesis process, photosystem II (PS-II), a Fe-containing protein complex, loses its activity because of low photosynthetic electron supply to Fe-starved plants^6,7^. Furthermore, the synthesis of Fe-S clusters and heme-containing proteins is severely affected in mitochondria of Fe deficient plants^8^. Thus, how Fe-deficiency affects Fe nutrition and growth is crucial for future genome editing strategies to improve plants.

A strategy-I plant acquires Fe by the reduction-based mechanism. In Strategy-I plants, ferric chelate reductase (FCR) is an enzyme that converts ferric ion to ferrous in roots to make existing Fe available^9^. This FCR activity and the upregulation of its candidate gene (*FRO*) have been mainly reported to confer Fe-deficiency tolerance in many dicot plants^5,10^. Also, the induction of acidification in the rhizosphere through proton (H^+^) extrusion enhances the mobilization of ferric Fe^11,12^. Although there have been few controversies on the role of phenolics increasing Fe availability in the rhizosphere, a few reports support that phenolics alter microbial community that in turn favors plant Fe uptake^13^.

Transporter genes are directly related to metal transport in plants. Further, the interactions of Fe with other mineral elements, such as zinc and sulfur, are often linked to the overall response of plants under Fe deficiency^4^. The most common Fe transporter is the *IRT1* gene (Fe-regulated transporter protein) that is known to transport ferrous Fe in several plants, including tomato^14^, field peas^15^, Arabidopsis^16^, etc. In plants, Fe and Zn homeostasis are complements to each other. *IRT* gene families are expressed in epidermal cells to mediate Zn transport in Fe-deficient roots^16,17^. Further, *Nramp1* (natural resistance-associated macrophage protein) also plays a role in Fe^2+^ transport in Fe-deprived plants^17,18^. Several ZIP proteins have been characterized in plants, usually showing modulation subjected to Fe/Zn deficiency^19^. The relation of Fe and S is of great importance in response to Fe deficiency as most active Fe in Fe-S protein clusters is tied to S in the chloroplast and cytochrome complex^20–23^. The molecular characterization of genes and their interactions linked to Fe uptake is still limited. Many critical biological pathways and gene families related to Fe uptake and transport remain unexplored in sunflower.

Sunflower (*Helianthus annuus* L.) is an important crop for edible oil production affected due to Fe-deficiency. However, the responses of Fe-deficient sunflower are not yet studied. Therefore, we investigated how Fe deficiency lessens growth and development in sunflower plants. Along with the morphophysiological evidence, a broad range of cellular and molecular responses were studied that trigger deficiency symptoms in Fe depleted sunflower. We further performed in silico analysis of Fe-related genes of sunflower to interpret the motifs, regulatory networks, and association of genes linked to Fe-deficiency.

## Materials and methods

### Plant cultivation system

Seeds of sunflower were sterilized with 70% ethyl alcohol for 3 min, followed by distilled water washing. The seeds were then germinated at room temperature for 2 days on a tray before transferring to solution culture (pH 6.0) in a 5 L plastic pot as previously described^24,25^. Sunflower seedlings were supplemented with two different concentrations of ferric Fe-EDTA (ethylenediaminetetraacetic acid): 25 µM Fe-EDTA (+Fe) and 1.0 µM Fe-EDTA (−Fe). The seedlings were cultivated in the growth cabinet having a 14/10 h light/dark photoperiod (550–560 µmol s^−1^ per µA). Plants were harvested for analysis after 2 weeks.

### Morphological and photosynthesis features

A digital caliper (Neiko 01407A Electronic Digital Caliper, United States) was used to measure the length of the roots and shoots. The dry weight of roots and shoots was taken after drying for 3 days at 80 °C in an electric oven. The leaf chlorophyll score was measured on a young leaf by a SPAD meter (Minolta, Japan). Furthermore, photosynthesis biophysics through chlorophyll fluorescence kinetic (OJIP), such as Fv/Fm (quantum efficiency of photosystem II), and Pi_ABS (photosynthesis performance index) were recorded on young leaves kept for 1 h in the dark using FP 100 PHOTON SYSTEM INSTRUMENT (CHECH REPUBLIC).

### Analysis of Fe and Zn concentration in roots and shoots

Briefly, fresh roots were washed with Milli-Q water and subsequently incubated at 4 °C in the first (10 mM MES) solution and in the second (10 mM MES + 1 mM EDTA), followed by washing 2–3 times in Milli-Q water. After surface cleaning, roots and shoots were kept on a falcon tube, keeping the lid open to dry at 70 °C for 3 days. Dried samples were subsequently digested with HNO_3_/HClO_4_ (3:1 v/v) and made volume up to 10 ml. The solution was then used for elemental analysis based on standard curves by Atomic absorption spectroscopy (SHIMADZU, JAPAN).

### Analysis of stress indicators

The standard curve for bovine serum albumin (BSA) was generated to estimate the total soluble protein, according to the Bradford assay^26^. Briefly, protein extraction was carried out by grinding the tissue samples with tris–HCL-buffer (50 mM, pH 7.5), 0.04% (v/v) β-mercaptoethanol, and 2 mM EDTA. The crude samples were centrifuged for 10 min at 12,000 rpm. The transparent fluid part was then mixed with 1 ml of Coomassie Brilliant Blue (CBB) before measuring the absorbance at 595 nm (60S UV–Visible Spectrophotometer, THERMO SCIENTIFIC, UNITED STATES). The concentration of unknown samples was then calculated based on the standard curve of different concentrations of BSA.

The electrolyte leakage demonstrating the loss of the cell membrane integrity were analyzed by a conductivity meter (HI98303, HANNA, UNITED STATES)^27,28^. Surface components of roots and shoots were dispensed frequently with deionized water. Thereafter, the freshly harvested samples were transferred into a beaker filled with 20 ml deionized water and kept at 25 °C for 2 h. Later, the solution’s electrical conductivity (EC1) was calculated. Afterward, the samples were heated in a water bath for 20 min at 95 °C then soothed at 25 °C before recording the final electrical conductivity (EC2). The electrolyte leakage was then determined as follows: = (EC1/EC2) × 100 (%).

The cell death percentage was estimated using Evans blue (SIGMA-ALRICH, UNITED STATES)^29,30^. The freshly harvested root and shoot were washed with MilliQ water. The tissue samples were then incubated in 2 ml Evan's blue mixture for 15 min at room temperature. Afterward, 1 ml of the 80% ethanol was added to the mixture and incubated for 10 min at room temperature. The tubes with solutions were then incubated for 15 min at 50 °C in a water bath and then centrifuged for 10 min at 12,000 rpm. The absorbance of the supernatant was recorded at 600 nm. Finally, the percentage of cell death in the root or shoot tissue was evaluated based on sample fresh weight.

### Fe chelate reductase activity

Fe (III)-FCR activity was determined in roots by ferrozine testing^5^. Briefly, the roots were washed once with 0.2 mM CaSO_4_ and 2–3 times with Milli-Q water to eliminate the surface contaminants. The root samples were then homogenized with 1 ml of assay mixture (100 mM Fe(III) EDTA, 0.10 mmol MES-NaOH (pH 5.5), 300 mM ferrozine). The samples and blank tubes (without assay mixture) were incubated in the dark for 20 min at 25 °C. Finally, aliquots were read at 562 nm. The FCR activity was determined by using the ferrozine molar extinction coefficient (1.50 × 103 M^−1^ cm^−1^).

### Estimation of rhizosphere acidification

The secretions of H^+^ from roots is known as proton extrusion. Briefly, the pH of the cultivation medium was maintained by 0.1 M HCl or 0.1 M KOH by a digital pH meter. The H^+^ efflux was measured by calculating the titrated quantity of acid or base to return pH to its starting point as follows: (amount of acid × concentration of acid) ÷ (fresh weight × time)^15^.

### Estimation of total phenolics

The total phenolics concentration in roots was measured as previously described^29,30^. In short, the root extracts were mixed with 80% of Folin-Ciocalteu reagent and the solution of 20% of Na_2_CO_3_. The optical density of the solution was read at 765 nm. The unknown samples were calculated on the basis of the gallic acid calibration curve.

### The qRT-PCR analysis

The total RNA was isolated from the fresh roots as described by SV total RNA isolation system (Promega, USA). The quantified RNA was then converted to cDNA using the cDNA synthesis kit (Promega, USA) before performing real-time PCR analysis in an Eco real-time PCR system (ILLUMINA, UNITED STATES) using gene-specific primers (Supplementary Table S1). The PCR reactions were set as follows: 95 °C for 3 min, followed by 40 cycles at 95 °C for 10 s, 56 °C for 30 s. The relative expression of candidate genes was calculated considered *Actin* as an internal control by the dd −∆Ct method^31^.

### Bioinformatics analysis

NCBI Blast program was run to retrieve the mRNA and protein sequences of HaIRT1, HaNramp1, HaZIP1, and HaFRO2. The CELLO (http://cello.life.nctu.edu.tw) server predicted the subcellular localization of proteins^27,28^. The MEGA (V. 6.0) developed the phylogenetic tree with the maximum likelihood (ML) method for 100 bootstraps using 11 HMA3 homologs from 11 plant species^32^. Furthermore, the ten conserved protein motifs of the proteins were characterized by MEME Suite 5.1.1 (http://meme-suite.org/tools/meme) with default parameters, but five maximum numbers of motifs to find^33^. The interactome network of HMA3 protein was generated using the STRING server (http://string-db.org) visualized in Cytoscape^34^. The PlantCare was used for scanning of *cis*-elements present in promoter regions of these genes^35^.

### Statistical analysis

At least three independent biological replications were considered for each sample in a randomized block design. The significance between +Fe and –Fe conditions was tested by t-test in Microsoft Excel 2007. In preparing graphic figures, GraphPad Prism 6 was used.

### Ethical approval and permission

Formal ethical approval is not required for this experimental work as the sunflower line used in this work is a cultivated genotype. In addition, the seeds were collected from the local market; hence, permissions and/or licences for collection of seed specimens are not required complying with relevant institutional, national, and international guidelines and legislation.

## Results

### Plant growth, photosynthetic efficiency and elemental concentration

**Figure 1:**
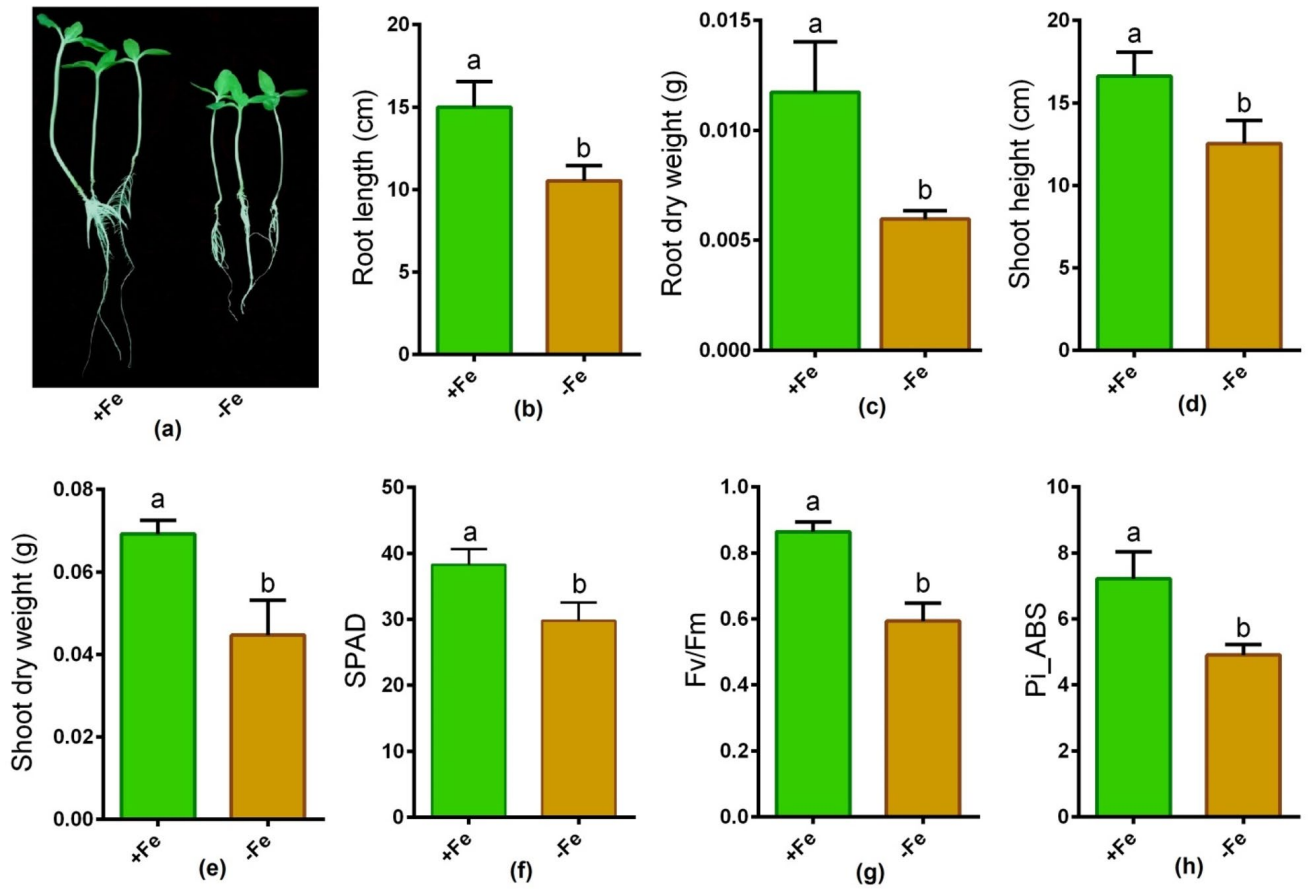
Plant phenotype (**a**), root length (**b**), root dry weight (**c**) shoot height (**d**), shoot dry weight (**e**), SPAD (**f**), Fv/Fm (**g**) and Pi_ABS (**h**) in sunflower cultivated under Fe-sufficient and Fe-deficient conditions. Different letters in each column indicate significant differences between means ± SD of treatments (n = 3) at a P < 0.05 significance level.

Along with the visual evidence, root and shoot morphological features (dry weight and length) significantly decreased owing to Fe-deficiency in the hydroponic solution compared to Fe-sufficient sunflower plants (Fig. 1a–e). In addition, the parameters associated with photosynthesis, such as SPAD score, Fv/Fm ratio, and Pi_ABS in young leaves, significantly declined due to Fe depletion relative to Fe-sufficient sunflower plants (Fig. 1f–h). Results showed that Fe and Zn concentration significantly reduced in roots and shoots under Fe-deficiency compared to Fe-sufficient controls (Table 1).

**Table 1:** Determination of Fe and Zn concentrations (µg g^−1^ DW) in roots and shoots of sunflower cultivated under Fe-sufficient and Fe-deficient conditions.

Nutrients	Roots			Shoots		
	+Fe	−Fe	Fold change	+Fe	−Fe	Fold change
Fe	116.8 ± 13.4^a^	70.5 ± 16.6^b^	1.6 fold	54.9 ± 10.1^a^	38.4 ± 9.2^b^	1.4 fold
Zn	132.5 ± 14.1^a^	63.7 ± 15.4^b^	2.0 fold	63.2 ± 4.7^a^	30.7 ± 4.6^b^	2.0 fold

Data represent means ± SD of three independent biological samples. Different letters indicate significant difference at P < 0.05 level.

### Changes in stress indicators

**Figure 2:**
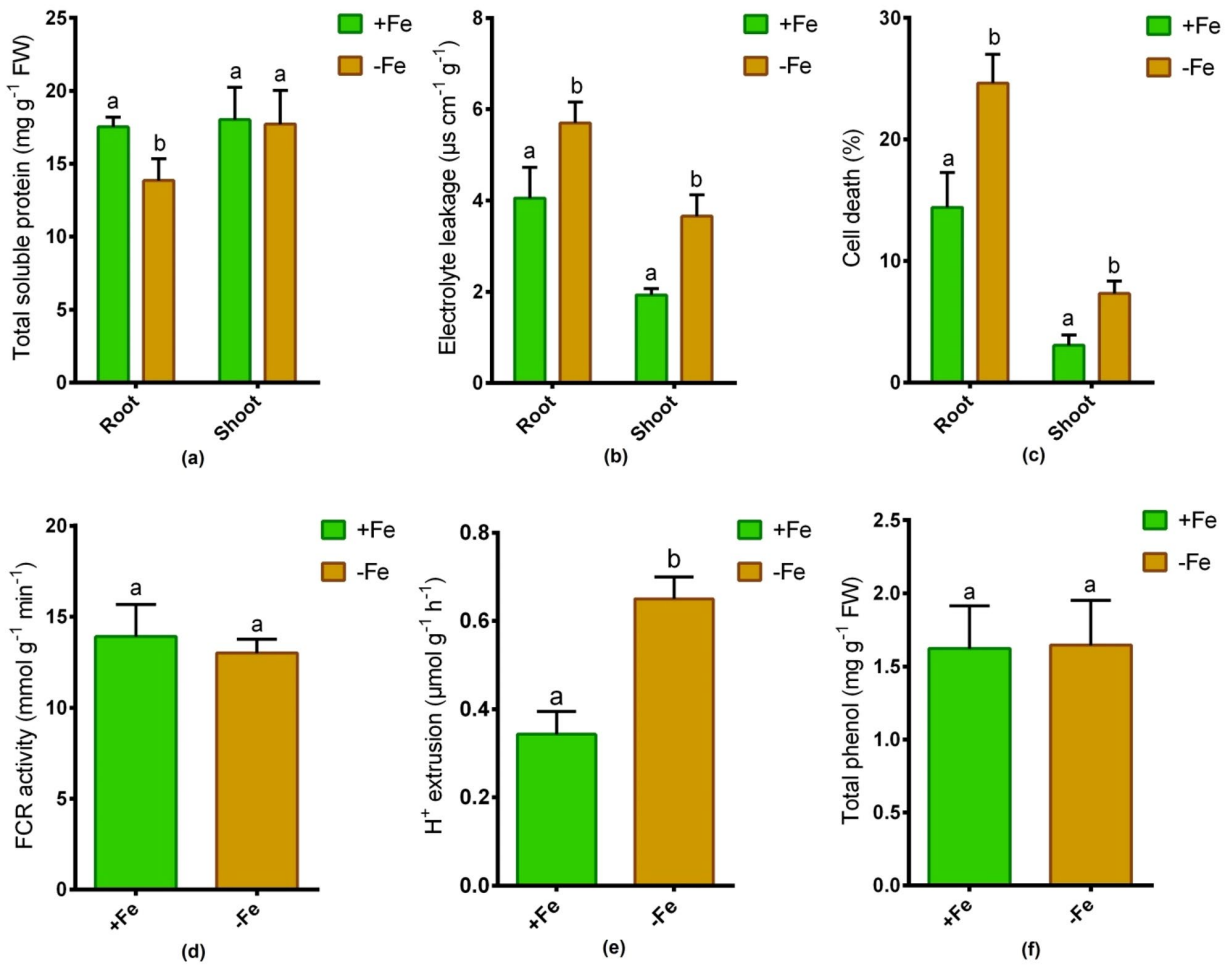
Total soluble protein (**a**), electrolyte leakage (**b**), cell death % (**c**), root FCR activity (**d**), root H^+^ extrusion (**e**) and root total phenol (**f**) in sunflower cultivated under Fe-sufficient and Fe-deficient conditions. Different letters in each column indicate significant differences between means ± SD of treatments (n = 3) at a P < 0.05 significance level.

Fe-deficiency is known to induce stress in plants. In this study, the total soluble protein concentration in roots significantly reduced, although it remained unchanged in shoots due to Fe-deprivation compared to Fe-sufficient controls (Fig. 2a). Fe-depletion demonstrated a significant enhancement in electrolyte leakage and cell death (%) in both roots and shoots relative to controls (Fig. 2b,c).

### Changes in strategy I responses in roots

Fe-depletion caused no significant changes in root FCR activity in comparison with Fe-sufficient plants (Fig. 2d). In contrast, the proton extrusion activity in roots was significantly induced owing to Fe-shortage in comparison with Fe-sufficient plants (Fig. 2e). On the other hand, total phenolics secretion in roots remained unchanged between Fe-sufficient and Fe-deficiency controls (Fig. 2f).

### Changes in the expression of Fe transporter and Strategy I genes

**Figure 3:**
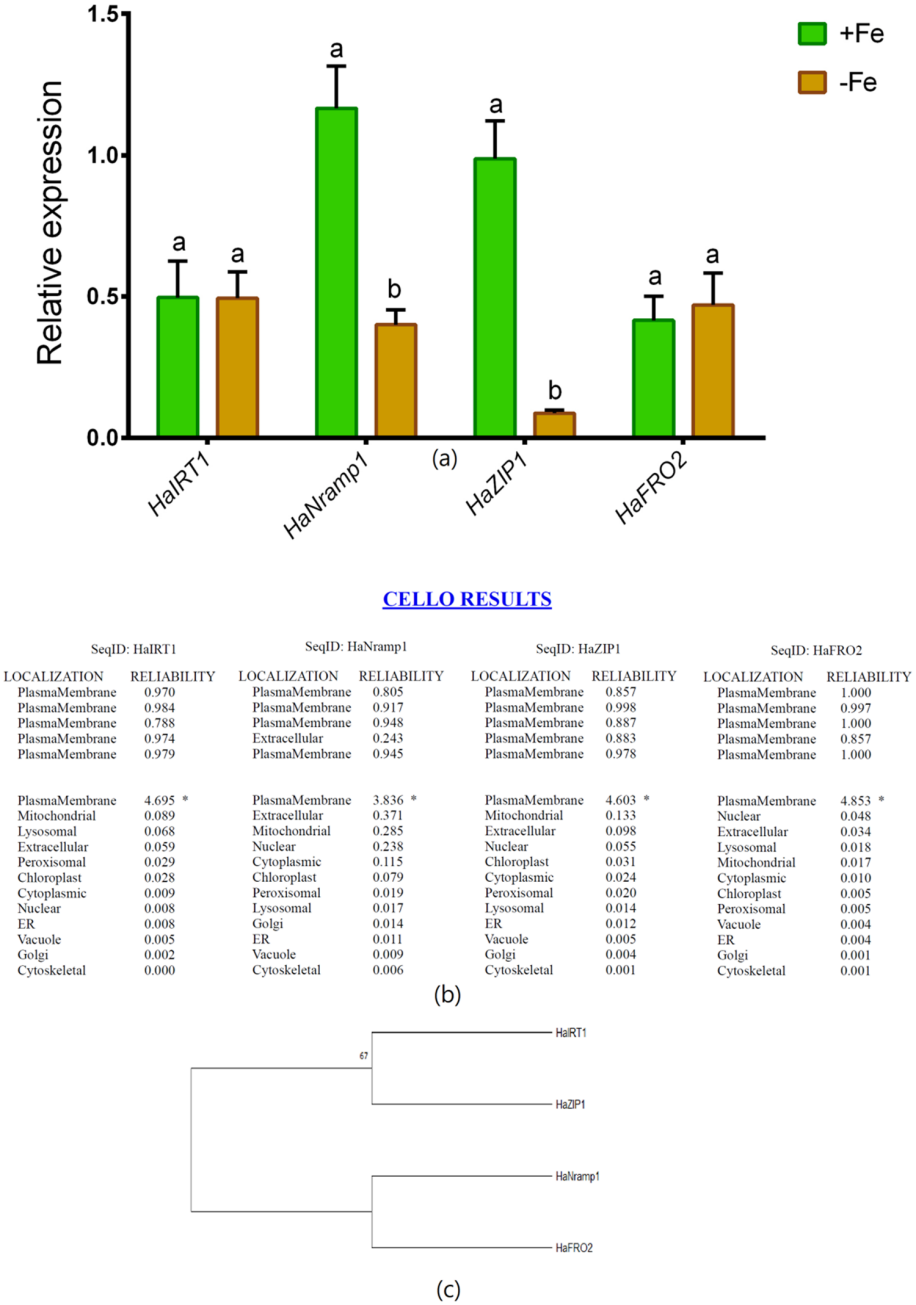
Quantitative expression (**a**), CELLO subcellular localization prediction (**b**) and phylogenetic tree of Fe-related genes (**c**) in roots of sunflower cultivated under Fe-sufficient and Fe-deficient conditions. Different letters in each column indicate significant differences between means ± SD of treatments (n = 3) at a P < 0.05 significance level. Trees were constructed by MEGA 6 software with the maximum likelihood (ML) method for 100 bootstrap values.

The expression of the *HaIRT1* gene remained unchanged under Fe-depletion relative to Fe-sufficient controls (Fig. 3a). However, *HaNramp1* and *HaZIP1* were significantly downregulated in roots owing to Fe-deprivation in comparison with Fe-sufficient plants (Fig. 3a). However, the expression of *HaFRO2* showed no substantial changes between Fe-sufficient and Fe-deficient conditions (Fig. 3a). The CELLO localization predictor showed that corresponding proteins of these genes are localized in the plasma membrane of roots (Fig. 3b). According to the phylogenetic tree, *HaIRT1* and *HaZIP1* are clustered together, while *HaNramp1* and *HaFRO2* are placed within-cluster, although all these genes are originated from the same ancestor (Fig. 3c).

### In silico analysis of Fe-related genes/proteins

**Figure 4:**
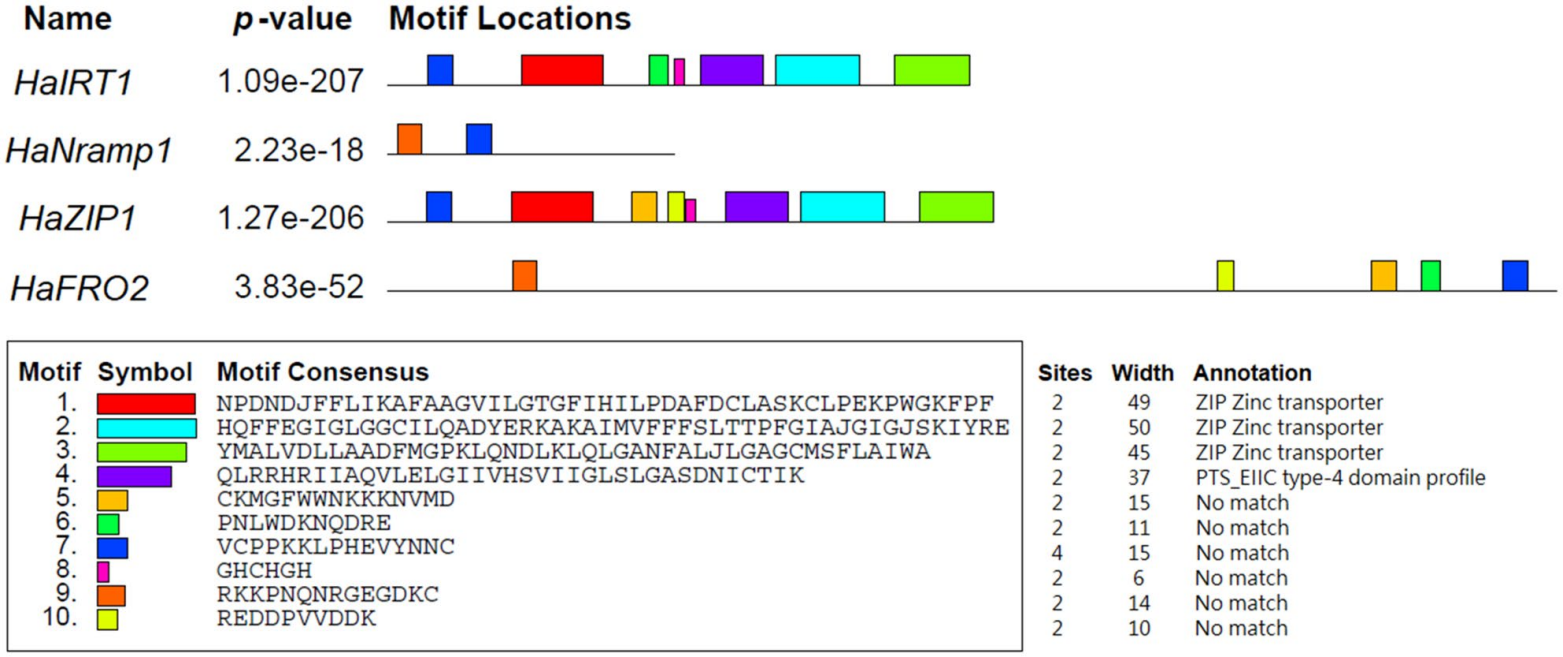
Schematic representation of the 10 conserved motifs in Fe-related proteins (*HaIRT1*, *HaNramp1*, *HaZIP1*, and *HaFRO2*). Scale bar corresponds to 0.1 amino acid substitution per residue. Different motifs, numbered 1–10, are displayed in different colored boxes.

MEME motif analysis tool searched for the most conserved motifs identified in *HaIRT1*, *HaNramp1*, *HaZIP1*, and *HaFRO2*. Most of these ten motifs are located at site two and contain 6–50 residues (Fig. 4). Four out of ten motifs matched to particular domains are as follows: motif 1 (NPDNDJFFLIKAFAAGVILGTGFIHILPDAFDCLASKCLPEKPWGKFPF), motif 2 (HQFFEGIGLGGCILQADYERKAKAIMVFFFSLTTPFGIAJGIGJSKIYRE), motif 3 (YMALVDLLAADFMGPKLQNDLKLQLGANFALJLGAGCMSFLAIWA), and motif 4 (QLRRHRIIAQVLELGIIVHSVIIGLSLGASDNICTIK). Motif 1, 2 and 3 correspond to ZIP zinc transporter, while motif 4 attaches PTS_EIIC type-4 domain profile (Fig. 4). Other motifs show no information (Fig. 4).

**Figure 5:**
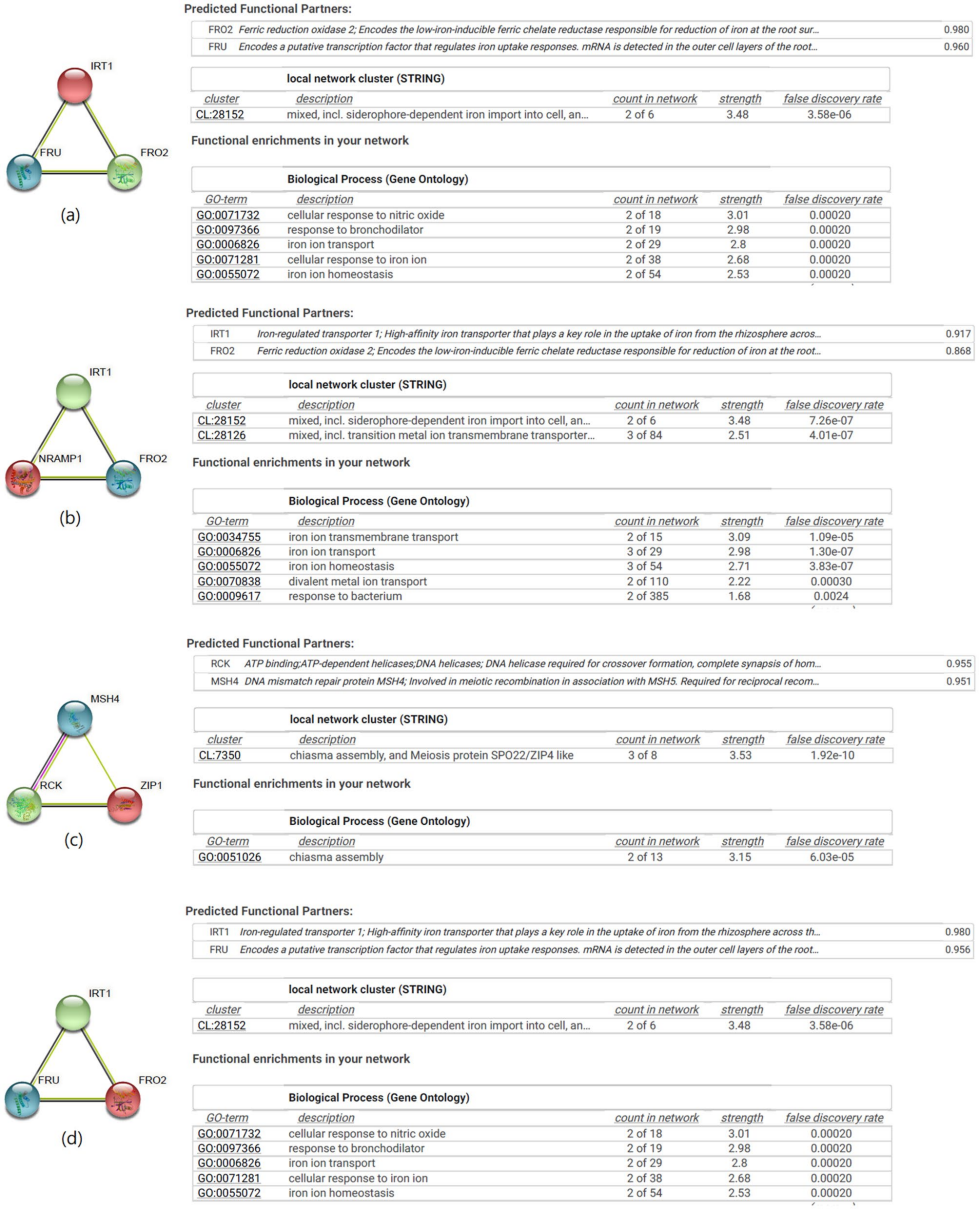
Gene interaction partners and gene network analysis of *HaIRT1* (**a**), *HaNramp1* (**b**), *HaZIP1* (**c**) and *HaFRO2* (**d**). Interactome was generated using Cytoscape for STRING data.

Interactome analysis was performed on the STRING server. The *HaIRT1* shows functional partnership with *FRO2* (ferric reduction oxidase 2) and *FRU* (a putative transcription factor) that regulates Fe uptake responses belonging to CL:28,152 local network cluster associated with siderophore-dependent Fe import into the cell (Fig. 5a). Functional enrichment of the network links to several biological functions, including cellular response to nitric acid, response to bronchodilator, Fe ion transport, cellular response to Fe ion, and Fe ion homeostasis. The *HaNramp1* links to *IRT1* (Fe-regulated transporter 1) and *FRO2* (ferric reductase oxidase 2) partners under two local network clusters (siderophore-dependent Fe import into the cell and transition metal ion transmembrane transporter). The *HaFRO2*′s partners are *IRT1* and *FRU* that regulated Fe uptake responses connected to CL:28,152 cluster (siderophore-dependent Fe import into the cell), having the same partner linked to the same biological processes observed for *HaIRT1* (Fig. 5d). This gene cluster is related to several biological processes, which include Fe ion transmembrane transport, Fe ion transport, Fe ion homeostasis, divalent metal ion transport, and response to a bacterium (Fig. 5b). Further, the functional partners of *HaZIP1* are *RCK* (ATP binding; ATP-dependent helicases; DNA helicases, etc.) and *MSH4* (DNA mismatch repair protein MSH4) under CL:7350 local cluster involved in chiasma assembly and meiosis protein (*SPO22/ZIP4* like) linked to the chiasma assembly biological process (Fig. 5c).

Versatile *cis*-regulatory elements correlated with studied genes were found through PlantCare tool. (Table 2). The *HaIRT1* promoter showed 1 ABRE (involved in the abscisic acid responsiveness), 1 ARE (essential for the anaerobic induction), 1 AuxRR-core (involved in auxin responsiveness), 8 CAAT-box (common elements in promoter and enhancer regions), 2 G-Box (involved in light responsiveness), 1 LTR (involved in low-temperature responsiveness), 1 MBS (MYB binding site involved in drought-inducibility), 1 RY-element (involved in seed-specific regulation), 2 TATA-box (core promoter element around − 30 of transcription start) and 1 TATC-box (involved in gibberellin-responsiveness). However, *HaNramp1* promoter showed 5 CAAT-box (element in promoter and enhancer regions), 6 TATC-box (involved in gibberellin-responsiveness), 1 CAT-box (related to meristem expression), 1 GATA-motif (part of a light-responsive element), and 1 HD-Zip 1 (involved in differentiation of the palisade mesophyll cells). The *HaZIP1* promoter revealed 1 ABRE (involved in the abscisic acid responsiveness), 1 ARE (essential for the anaerobic induction), 6 CAAT-box (promoter and enhancer regions), 23 TATA-box (core promoter element around − 30 of transcription start), 1 TATC-box (involved in gibberellin-responsiveness), 1 GATA-motif (part of a light-responsive element), 1 ATCT-motif (part of a conserved DNA module involved in light responsiveness), 4 Box 4 (part of a conserved DNA module involved in light responsiveness), 1 CGTCA-motif (involved in the MeJA-responsiveness), 1 O_2_-site (involved in zein metabolism regulation), 1 SARE (involved in salicylic acid responsiveness), 2 TCA-element (involved in salicylic acid responsiveness), 1 TGA-element (auxin-responsive element), 1 TGACG-motif (involved in the MeJA-responsiveness) and 1 chs-CMA2b (part of a light-responsive element). The *HaFRO2* promoter showed numerous *cis*-acting elements which include 1 ABRE (involved in the abscisic acid responsiveness), 6 ARE (essential for the anaerobic induction), 15 CAAT-box (promoter and enhancer regions), 2 G-Box (involved in light responsiveness), 1 LTR (involved in low-temperature responsiveness), 24 TATA-box (core promoter element around − 30 of transcription start), 2 TATC-box (involved in gibberellin-responsiveness), 1 CAT-box (related to meristem expression), 1 GATA-motif (part of a light responsive element), 1 HD-Zip 1 (involved in differentiation of the palisade mesophyll cells), 1 CGTCA-motif (involved in the MeJA-responsiveness), 1 O_2_-site (involved in zein metabolism regulation), 1 TGA-element (auxin-responsive element), 1 TGACG-motif (involved in the MeJA-responsiveness), 1 AACA_motif (involved in endosperm-specific negative expression), 1 AE-box (part of a module for light response), 1 AuxRR-core (involved in auxin responsiveness), 1 Box II (part of a light responsive element), 1 CCAAT-box (MYBHv1 binding site), 3 GT1-motif (light responsive element), 2 I-box (part of a light responsive element), 1 LAMP-element (part of a light responsive element), 3 P-box (gibberellin-responsive element), 2 TCT-motif (part of a light responsive element) and 1 circadian (involved in circadian control). CAAT-box (promoter and enhancer regions) and TATA-box (core promoter element around − 30 of transcription start) are shared among the four Fe-related genes of sunflower (Table 2).

**Table 2:** *Cis*-regulatory element analysis of *HaIRT1, HaNramp1, HaZIP1 and HaFRO2* promoters.

Genes	ABRE	ARE	AuxRR-core	CAAT-box	G-Box	LTR	MBS	RY-element	TATA-box	TATC-box	CAT-box	GATA-motif	HD-Zip 1	ATCT-motif	Box 4	CGTCA-motif	
*HaIRT1*	1	1	1	8	2	1	1	1	2	1							
*HaNramp1*				5					6		1	1	1				
*HaZIP1*	1	1		6					23	1		1		1	4	1	
*HaFRO2*	1	6		15	2	1			24	2	1	1	1			1	

Genes	O^2^-site	SARE	TCA-element	TGA-element	TGACG-motif	chs-CMA2b	AACA_motif	AE-box	AuxRR-core	Box II	CCAAT-box	GT1-motif	I-box	LAMP-element	P-box	TCT-motif	Circadian
*HaIRT1*																	
*HaNramp1*																	
*HaZIP1*	1	1	2	1	1	1											
*HaFRO2*	1			1	1		1	1	1	1	1	3	2	1	3	2	1

ABRE (involved in the abscisic acid responsiveness), ARE (essential for the anaerobic induction), AuxRR-core (involved in auxin responsiveness), CAAT-box (located in promoter and enhancer regions), G-Box (involved in light responsiveness), LTR (involved in low-temperature responsiveness), MBS (MYB binding site involved in drought-inducibility), RY-element (involved in seed-specific regulation), TATA-box (core promoter element around − 30 of transcription start), TATC-box (gibberellin-responsiveness), CAT-box (related to meristem expression), GATA-motif (part of a light responsive element), HD-Zip 1 (involved in differentiation of the palisade mesophyll cells), ATCT-motif (part of a conserved DNA module involved in light responsiveness), Box 4 (part of a conserved DNA module involved in light responsiveness), CGTCA-motif (involved in the MeJA-responsiveness), O_2_-site (involved in zein metabolism regulation), SARE (involved in salicylic acid responsiveness), TCA-element (involved in salicylic acid responsiveness), TGA-element (auxin-responsive element), TGACG-motif (involved in the MeJA-responsiveness), chs-CMA2b (part of a light responsive element), AACA_motif (involved in endosperm-specific negative expression), AE-box (part of a module for light response), Box II (part of a light responsive element), CCAAT-box (MYBHv1 binding site), GT1-motif (light responsive element), I-box (part of a light responsive element), LAMP-element (part of a light responsive element), P-box (gibberellin-responsive element), TCT-motif (part of a light responsive element), circadian (involved in circadian control).

## Discussion

### Plant growth and photosynthesis under Fe deficiency

Although Fe-deficiency causes damages in plants, a clear understanding of this nutritional stress in sunflower was hazy. In this study, Fe deficiency caused a severe reduction in length and biomass of roots and shoots in sunflower. In addition, the SPAD score dramatically dropped due to Fe starvation, suggesting that chlorophyll degradation in sunflower leaves are associated with the damages in photosynthetic apparatus due to Fe-deficiency. This was further supported by the decrease in the efficiency of photosystem II and photosynthesis performance index in Fe-starved leaves of sunflower. The reduction in the quantum yield of photosystem II is often associated with the Fe-deficient leaves^36^. Several studies also documented that a chlorotic leaf is closely correlated with photosystem II efficacy in plants under Fe deficiency^37,38^. A study demonstrated that the redox state of photosystem II acceptors was negatively affected due to Fe deficiency in sugar beet^39^. Moreover, proteins linked to the reaction center and light-harvesting antenna usually decline in Fe-starved leaves^40^. It implies that Fe-deficiency appears to inhibit the uptake of Fe but is also closely linked to photosynthetic inefficiency in sunflower. Furthermore, the changes in photosystem II parameters due to Fe-deficiency may be linked to damage in the reaction center or various elements of the energy transfer path in the photosystem II system in Fe-deficient sunflower plants. This message can be useful to strengthen the knowledge to avoid damage to the photosynthetic machinery in sunflower.

### Changes in Fe concentration and transporter genes

In this study, the *HaIRT1* showed no changes in roots owing to Fe-deficiency, suggesting that this transporter is possibly not involved in Fe-deficiency tolerance in sunflower. In plants, a dual pattern of *IRT1* expression was reported in Fe-depleted Arabidopsis^41^. Thus, it may be possible that the expression is highly dependent on the genotypic background of the cultivar/species and the duration of stress exposed to plants. In contradiction to the expression studies on Fe transporters, studies showed that the expression of *IRT1* was induced in Fe-starved tomato^42^. Another study showed the role of *Nramp1* in Fe mobilizing^42^, but it also cooperates with *IRT1* to take up Fe in response to Fe-deficient conditions in Arabidopsis^43^. In this study, the *HaNramp1* significantly downregulated in roots due to Fe-deprivation, suggesting that *HaNramp1* is directly associated with the decreased Fe uptake along with its long-distance transport of Fe that eventually resulted in severe growth reduction and photosynthesis damages in sunflower. Also, ZIP proteins are involved in the uptake and transport of Fe and Zn in plants^14,16^. This is very much consistent with the decrease in Zn concentration and *HaZIP1* expression in Fe-deficient sunflower roots. In general, the expression of *ZIP* genes is induced when plants get deficient in Zn, which facilitates cell Zn influx and Zn movement between organisms and also when plants become deficient in Fe or Mn^44,45^. Our results imply that *HaZIP1* is also involved, at least in part, with Fe acquisition or fully dedicated to Zn uptake in sunflower plants or highly interacting with the Fe status of the sunflower plants. Furthermore, Fe-deficiency is also occurred due to excessive manganese in plants, inhibiting photosynthesis^46^. Consequently, photoinhibition of PSII may be the ultimate consequence of Mn exposure^47^. We, therefore, suggest that Fe-deficiency-induced reduction of Fe is tightly linked with the status of other essential elements, leading to the overall sensitivity to stress in sunflower plants. Moreover, the decreased Fe uptake and subsequent translocation largely attributed to the downregulation of several transporters (*HaNramp1* and *HaZIP1*) involved in the uptake of Fe and Zn in roots, which agree with the severe chlorosis and photosynthesis damage in Fe-depleted sunflower.

### Changes in Strategy I responses

In this study, we assessed the status of Strategy I responses in Fe-deprived sunflower plants. The FCR activity and its responsible *HaFRO2* gene were consistently stable following Fe-starvation in the roots of sunflower. The constitutive expression or upregulation of the *FRO1* in Fe-deficient plants, was reported in other plants^48,49^. However, the variations in FCR activity in response to Fe starvation were also reported in several Strategy I plants^15,50^. Studies also underscored the importance of FCR in Fe metabolism and photosynthetic efficiency in plants^51^. Not only that, the degradation of soluble proteins and the occurrence of the cell membrane damages in plants are often related to Fe-deficiency^5^, which was also evident in Fe depleted sunflower. Taken together, our findings confirm that the induction of FCR is a key factor in withstanding Fe-deficiency in sunflower. Thus, the expression *HaFRO1* is one of the strategies that can be modulated to improve Fe-efficiency in sunflower.

Computational prediction suggests that *HaIRT1*, *HaNramp1*, *HaZIP1,* and *HaFRO2* genes are localized in the plasma membrane. The *Nramp* and *ZIP* transporters are mainly distributed in the plasma membrane in mulberry^52^. Besides, *IRT1* located in the plasma membrane has been established as one of the key plant model proteins for studying Fe acquisition and endomembrane trafficking^53^. We also constructed a phylogenetic tree in which *HaIRT1* and *HaZIP1* are clustered together while *HaNramp1* and *HaFRO2* are located in another cluster, but all these four genes are originated from the same evolutionary ancestor. The coordination of Fe and Zn transporters are widely known in Fe-deficient plants because of the similarities of these metal elements^54^.

Fe becomes unavailable to plants if the cultivation conditions have a pH higher than 8.0^15^. As a result, plants release H^+^ in the rhizosphere to reduce the pH level^5,51^. This is a common Strategy I trait, enabling Fe acquisition that showed a substantial increase as evident from the proton extrusion activity in roots of sunflower. The proton extrusion involvement in Fe-depleted conditions was reported in other dicot plants^15,55^. Although the proton extrusion can contribute to Fe uptake of sunflower, this adaptive trait was a game-changer to maintain the overall Fe uptake system in Fe-deprived sunflower. Moreover, overcoming Fe-deficiency-induced is a complex process involving cumulative biochemical and molecular induction of Strategy I responses in sunflower plants. Further, the phenolic compounds released by roots responsible for Fe-chelating under Fe deficiency, as previously reported^56,57^, appear to be not involved with Fe homeostasis in sunflower plants.

### In silico characterization of Fe-related genes

In recent years, in silico characterization of candidate genes before the wet-lab experiment is routinely performed to narrow down the target of studies. Conserved motifs are identical sequences of plant species that are maintained by natural selection. A highly conserved sequence has functional roles in plants and can be a useful start point for researching a particular topic of interest^58^. In this study, three motifs of *HaIRT1* and *HaZIP1* proteins are linked to *ZIP* zinc transporter. Several members of the Zn-regulated transporters and Fe-regulated transporter-like Protein (ZIP) gene family have shown to be involved in metal uptake and transport^59^. In maize, ZIP proteins are localized in cells playing important roles in the uptake of divalent ions^60^. Also, several ZIP genes are highly induced in roots and are involved in Zn uptake under Zn deficient conditions in barley^61^ and bean^62^. In the light of these findings, it suggests that sunflower ZIP transporter might play a role in Fe and Zn uptake and distribution under low Fe availability.

The interaction network of a specific gene provides information about associations that may affect the regulations in response to particular stress in plants. The interactome map analyzed in the String platform showed a close partnership of *FRO2* and *FRU* genes with sunflower with *HaIRT1*, *HaNramp1*, *HaZIP1*, and *HaFRO2* genes generally linked to Fe uptake system in plants. Mutant studies showed that plants overexpressing *FRO2* grew at a much higher rate than wild-type during Fe-deficient conditions^63^. Besides, the *FRU* gene is a mediator in inducing iron mobilization responses, indicating that iron uptake regulation is preserved in dicot species^64^. Thus, the biological functions of these sunflower Fe-related genes are involved in Fe ion transport and Fe ion homeostasis. Overall, this interactome finding might provide essential background for functional genomics studies of Fe uptake and transport in sunflower and related plant species.

Besides, promoter analysis reveals the involvement of *cis*-acting elements in *HaIRT1*, *HaNramp1*, *HaZIP1*, and *HaFRO2* commonly associated with *cis*-acting elements in promoter and enhancer regions (CAAT-box) along with core promoter element around − 30 of transcription start (TATA-box). Other than *HaNramp1*, the Fe-related genes of sunflower are predominantly linked to *cis*-acting elements involved in the abscisic acid responsiveness, gibberellin-responsiveness, GATA-motif (part of a light-responsive element) and anaerobic induction. Abscisic acid is known to contribute to the response to oxidative damage in *Arabidopsis thaliana*^65^. In this study, the HaIRT1 promoter is linked to auxin and salicylic acid elements, while HaZIP1 and HaFRO1 promoters are generally attached to methyl jasmonate-responsive elements in sunflower. Auxin signaling affects Fe signaling and the Fe deficiency response in plants^66^. A recent study revealed that auxin plays systemic action on the expression of *FRO1* in tomato^67^. Further, Fe deficiency induces salicylic acid signaling, thereby activating Fe translocation via the *bHLH38/39*-mediated transcriptional regulation of downstream Fe genes^68^. Gibberellin signaling controls the expression of transcription factor regulation iron-uptake machinery genes^69^, while jasmonate signaling is involved in the expression of Fe-deficiency induced genes in plants^70^. Studies demonstrated that promoter is anaerobically induced in plant tissues resulted in the synthesis of a new set of polypeptides or anaerobic stress proteins, but its connection with Fe-deficiency still needs to be established plants^71^. Altogether, our analysis implies that some hormones can be targeted to improve Fe-efficiency in sunflower.

## Conclusion

This study gives new insights into the mechanical basis for Fe-deficiency responses in sunflower. Fe deprivation caused severe physiological and photosynthetic damage in sunflower. Further Fe-deficiency in sunflower resulted in a simultaneous decrease in Fe and Zn status of the plants. This physiological evidence was further supported by the downregulation of *HaNramp1* and *HaZIP1* transcripts in Fe-starved sunflower roots. Fe-related genes (*HaIRT1* and *HaZIP1*) are localized in the plasma membrane and are predominantly linked to motifs linked to *ZIP* zinc transporter. Interestingly, FRO2 and FRU partners showed a close association with *HaIRT1*, *HaNramp1*, *HaZIP1*, and *HaFRO2* genes in sunflower in addition to the presence of *cis*-regulatory elements in promoters associated with auxin, salicylic acid, gibberellin, and methyl jasmonate-responsive elements. These results explore our understanding of Fe-starvation responses in the sunflower that can be further utilized in genome-editing or breeding programs to develop Fe-efficient genetic lines.

## Supplementary Information

Supplementary Information.
