# Supplementary material for: Molecular characterization of Fe-acquisition genes causing decreased Fe uptake and photosynthetic inefficiency in Fe-deficient sunflower
Source: Sci Rep. 2021 Mar 10;11:5537. doi: 10.1038/s41598-021-85147-z (PMC7947006; doi:10.1038/s41598-021-85147-z)
Supplement: Supplementary file 1 — Supplementary Information. [file 41598_2021_85147_MOESM1_ESM.docx]

**Molecular characterization of Fe-acquisition genes causing decreased Fe uptake and photosynthetic inefficiency in Fe-deficient sunflower**

**Ahmad Humayan Kabir^1*^, Sharaban Tahura^1^, Mona M. Elseehy^2^, Ahmed M. El-Shehawi^3^**

**Supplementary Table S1.** Primers sequences used for qPCR analysis.

| *HaActin*  *XM_022185683.2* | F | ATGAATCTGGGCCGTCCATC | 97 bp |
| --- | --- | --- | --- |
|  | R | TTTCGAACCCCAAATGGCAAC |  |
| *HaIRT1*  *XM_022113140.2* | F | CCATCCATCGTAGGGTTCCGT | 74 bp |
|  | R | TGGAGAGCAGGAGGAGAGAG |  |
| *HaNramp1*  *XM_022165885.2* | F | ATCTCCCGCTCTTCTCAAAAT | 86 bp |
|  | R | AGGAATCGAGCGGAGAGAGA |  |
| *HaZIP1*  *XM_022173172.2* | F | CCGGATGCTTTCGACCATCT | 94bp |
|  | R | GCGATAAATCCTGCGAACGG |  |
| *HaFRO2*  *XM_022180093.2* | F | GCTAACTTCTGAGGCCAGCA | 175 bp |
|  | R | CAAAGCCAACTCTCCGGCTA |  |
